# P08 Vanishing bile duct syndrome in haemophagocytic lymphohistiocytosis precipitated by Stevens-Johnson syndrome: a rare case report

**DOI:** 10.1093/rap/rkae117.039

**Published:** 2024-11-05

**Authors:** Gagandeep Sukhija, Christopher Baldwin, Eman Elfar, Jamie Thoroughgood, Andrew Rutherford, Chris Wincup, Arti Mahto

**Affiliations:** King’s College Hospital NHS Foundation Trust, London, United Kingdom; King’s College Hospital NHS Foundation Trust, London, United Kingdom; King’s College Hospital NHS Foundation Trust, London, United Kingdom; King’s College Hospital NHS Foundation Trust, London, United Kingdom; King’s College Hospital NHS Foundation Trust, London, United Kingdom; King’s College Hospital NHS Foundation Trust, London, United Kingdom; King’s College Hospital NHS Foundation Trust, London, United Kingdom

## Abstract

**Introduction:**

Vanishing bile duct syndrome (VBDS) is a rare, acquired disease presenting with features of cholestasis and loss of bile ducts (>50%) on biopsy. Haemophagocytic lymphohistiocytosis (HLH) is a life-threatening autoinflammatory disorder characterised by excessive activation of the immune system, leading to severe inflammation, pancytopenia, and multi-organ failure. Both can be associated with multiple factors including neoplastic, infectious, autoimmune or medications. However, concurrence of HLH and VBDS have been reported rarely in the literature. We present a rare case of HLH complicated by VBDS, with Steven-Johnson syndrome (SJS) as a precipitant. To our knowledge, this a third case of such association.

**Case description:**

A previously healthy 15-year-old girl was admitted for tertiary liver support due to severe liver dysfunction. Her symptoms began after an episode of pharyngitis treated with antibiotics and anti-inflammatories. On day three, she developed an erythematous and desquamative rash starting on her cheeks and spreading to her entire body. Diagnosed with SJS, she received supportive care locally.

She re-presented to her local hospital with worsening pruritus, diarrhoea, jaundice, and abdominal pain. Blood tests showed cholestatic liver dysfunction (AST 350 U/L, bilirubin 400 µmol/L, alkaline phosphatase 780 U/L). Her condition worsened with fever and haemodynamic instability, prompting ITU admission. She developed progressive pancytopenia (Hb 68, Plt 70 x10^9/L, WCC 0.6 x10^9/L), necessitating transfusions and GCSF. Elevated ferritin (73,000 µg/L) and triglycerides raised suspicion of HLH.

A bone marrow examination confirmed HLH, while a liver biopsy, prompted by persistently elevated liver enzymes, showed extensive bile duct loss suggestive of VBDS without HLH activity. Her autoimmune serology was positive for Ro52/60, though its significance was unclear. Imaging, including a CT scan and later a PET scan, ruled out malignancy.

She was treated aggressively with steroids and plasma exchange, followed by IVIG, tocilizumab, anakinra, and cyclosporine. This normalised her cell counts (Hb 84, Plt 70 x10^9/L, WCC 9 x10^9/L), with a repeat BM biopsy showing reduced HLH activity. Her liver function (AST 650 U/L, bilirubin 400 µmol/L, alkaline phosphatase 3000 U/L) and ferritin (30,000 µg/L) remained elevated. Soluble interleukin-2 was used as a surrogate marker for HLH activity due to the possibility of ferritin levels being affected by concomitant liver injury.

She is subsequently managed with mycophenolate and tacrolimus, improving her HLH markers, though her liver functions remain abnormal. Following MDTs, she is now awaiting combined liver and bone marrow transplants.

**Discussion:**

Cases of Stevens-Johnson Syndrome complicated by HLH have been reported in the literature, but HLH in association with VBDS is extremely rare. Li et al. reported a case of VBDS triggered by amoxicillin, complicated by HLH, which required aggressive management. Similarly, Lin et al. documented a case of VBDS complicated by HLH and SJS, where the patient unfortunately succumbed to secondary infections. Our case, likely precipitated by SJS, is exceptionally rare and possibly the third reported instance of such a combination. The liver biopsy in our case did not show evidence of HLH but was consistent with the loss of bile ducts, characteristic of VBDS.

The management of HLH is typically very aggressive, involving plasma exchange, IVIG, anakinra, or drugs like etoposide, depending on the presentation and underlying cause. In our patient, anakinra resulted in severe mouth ulcers and there was a concern about using tocilizumab in the context of severe hepatic dysfunction. We used soluble interleukin-2 (sIL-2) as a surrogate marker for HLH activity in our case as it was unclear whether the persistently raised ferritin was arising from ongoing liver dysfunction, cholestasis or recurrent transfusions necessitating.

Managing this case required multiple multidisciplinary team (MDT) meetings involving paediatric, haematology, hepatology, and rheumatology specialists.

For our patient, liver transplantation, which is the mainstay of treatment for progressive liver dysfunction, has been delayed due to the coexisting ongoing HLH, which increases the risk of transplant failure. While her HLH markers have improved with mycophenolate and tacrolimus, she is due repeat liver and bone marrow biopsies to ascertain the activity of HLH and for preparation for combined liver and bone marrow transplant. Her clinical course has been complicated by infections along with flares and relapses of HLH.

**Key learning points:**

• The initial presentation of SJS following an episode of pharyngitis treated with antibiotics highlights the need for vigilance in recognising drug reactions and their potential to precipitate severe systemic diseases like HLH.

• Persistent liver failure in HLH, especially with cholestatic picture, should raise the possibility of concomitant VBDS.

• Management of overlapping syndromes like SJS, HLH, and VBDS requires a coordinated effort and MDT approach involving haematologists, rheumatologists, hepatologists, and intensivists to tailor treatment strategies effectively.

• Prompt initiation of immunosuppressive therapies, including IVIG, corticosteroids, and immunomodulators like anakinra, is essential in controlling the hyperinflammatory state of HLH.

• The use of sIL-2 as a surrogate marker of inflammation in the presence of severe liver dysfunction.

• Clear communication, family support and allied health professional involvement is also key in such cases that carry high mortality and result in a protracted hospital admission.

• A plausible complex, causal relationship between SJS, HLH and VBDS, which may involve immune mediated response to infection or a drug reaction.

This case underscores the complexity and rarity of managing concurrent SJS, HLH, and VBDS, highlighting the importance of a tailored, multidisciplinary approach and the need for novel strategies for management.

